# Impact of the *Monocarboxylate Transporter-1* (MCT1)-Mediated Cellular Import of Lactate on Stemness Properties of Human Pancreatic Adenocarcinoma Cells †

**DOI:** 10.3390/cancers12030581

**Published:** 2020-03-03

**Authors:** Leontine Sandforth, Nourhane Ammar, Lisa Antonia Dinges, Christoph Röcken, Alexander Arlt, Susanne Sebens, Heiner Schäfer

**Affiliations:** 1 Institute for Experimental Cancer Research—Christian-Albrechts-University & UKSH Campus Kiel-Bldg. U30, Arnold-Heller-Straße 3, 24105 Kiel, Germany; leontine.sandforth@yahoo.de (L.S.); nourhane.h.ammar@gmail.com (N.A.); lisa_dinges@gmx.de (L.A.D.); susanne.sebens@email.uni-kiel.de (S.S.); 2 Department of Pathology—Christian-Albrechts-University & UKSH Campus Kiel-Bldg. U33, Arnold-Heller-Straße 3, 24105 Kiel, Germany; christoph.roecken@uksh.de; 3 Biomaterial Bank of the Comprehensive Cancer Center Kiel—UKSH Campus Kiel-Bldg. 17, Arnold-Heller-Straße 3, 24105 Kiel, Germany; 4 Department of Internal Medicine I-UKSH Campus Kiel-Bldg. K3, Arnold-Heller-Straße 3, 24105 Kiel, Germany; aarlt@1med.uni-kiel.de

**Keywords:** tumor–stroma interactions, metabolic reprogramming, cancer stem cells, chemoresistance

## Abstract

Metabolite exchange between stromal and tumor cells or among tumor cells themselves accompanies metabolic reprogramming in cancer including pancreatic adenocarcinoma (PDAC). Some tumor cells import and utilize lactate for oxidative energy production (reverse Warburg-metabolism) and the presence of these “reverse Warburg“ cells associates with a more aggressive phenotype and worse prognosis, though the underlying mechanisms are poorly understood. We now show that PDAC cells (BxPc3, A818-6, T3M4) expressing the lactate-importer monocarboxylate transporter-1 (MCT1) are protected by lactate against gemcitabine-induced apoptosis in a MCT1-dependent fashion, contrary to MCT1-negative PDAC cells (Panc1, Capan2). Moreover, lactate administration under glucose starvation, resembling reverse Warburg co a phenotype of BxPc3 and T3M4 cells that confers greater potential of clonal growth upon re-exposure to glucose, along with drug resistance and elevated expression of the stemness marker Nestin and reprogramming factors (Oct4, KLF4, Nanog). These lactate dependent effects on stemness properties are abrogated by the MCT1/lactate-uptake inhibitor 7ACC2 or MCT1 knock-down. Furthermore, the clinical relevance of these observations was supported by detecting co-expression of MCT1 and reprogramming factors in human PDAC tissues. In conclusion, the MCT1-dependent import of lactate supplies “reverse Warburg “PDAC cells with an efficient driver of metabostemness. This condition may essentially contribute to malignant traits including therapy resistance.

## 1. Introduction

Pancreatic ductal adenocarcinoma (PDAC) contributes to a large proportion of mortality among all cancers in Western countries exhibiting a poor 5 year survival rate of less than 10% [1]. This is mainly due to the late diagnosis at an already advanced stage and the profound therapy resistance. Accordingly, much effort is still needed to better understand the early steps of PDAC development in order to identify molecules that can be used for screening, early diagnosis, chemoprevention, and/or targeted therapies. Precursor lesions of PDAC predominantly originate from ductal cells with pancreatic intraepithelial neoplasia (PanIN) being the most frequent and best characterized premalig­nant lesion of PDAC [2,3]. Besides early genetic alterations, in particular, the mutation of the onco­gene *kras* one hallmark of pancreatic carcinogenesis [4] is a pronounced stromal microenvironment comprising stellate cells, myofibroblasts, and diverse immune cells together with extracellular matrix [5,6,7]. Given the intense desmoplasia and the profound tumor stroma in PDAC [8], different traits in the metabolism of stroma and cancer cells substantially contribute to the tumor heterogeneity and greatly impact on the malignancy of the disease. Thus, the appearance and fate of cancer cells in such stroma enriched tumors may be governed by their differential or even reciprocal metabolism.

It is meanwhile widely accepted that alterations in the energy and glucose metabolism, termed metabolic reprogramming, belong to the cancer hallmarks. Indeed, cancer cells exhibit profound changes in metabolite utilization and formation that relate to malignant growth and progression [9,10]. While the observation that tumors produce high amounts of lactate dates back to the 1920s, the exact mechanisms by which an altered metabolism of cancer cells supports their malignant phenotype are still not fully understood. Originally designated as aerobic glycolysis or Warburg effect, many tumors consume amounts of glucose irrespective of oxygen supply [11]. Thus, proliferating tumor cells depend on glycolytic glucose utilization to drive biomass production [12,13], e.g., via the pentose–phosphate pathway (PPP) and cataplerosis from the Kreb’s cycle. For maintaining high-rate glycolysis, pyruvate needs to be reduced to lactate as waste product which, after its release by tumor cells, can also modulate the cellular microenvironment.

Another condition observed in many advanced cancers, however, manifests in the lactate-uptake by certain tumor cells [14,15,16]. Particularly under conditions of glucose restriction, these tumor cells utilize lactate for energy production and as anaplerotic substrate. In most cases, the lactate taken up by these tumor cells derives from surrounding stromal cells, such as fibroblasts, or from other tumor cells addicted to and consuming high amounts of glucose. In this fashion, metabolic symbiosis and energy transfer is maintained between stromal and tumor cells or between tumor cells themselves [17,18,19], a modality termed “reverse Warburg“ [20]. Recent studies revealed that reverse Warburg conditions are implicated in the progression and poor outcome of malignancies, e.g., breast, prostate, endometrial or colorectal cancer [21,22,23,24,25,26]. The lactate/proton symporter monocarboxylate transporter-1 (MCT1) and -4 (MCT4) have a key role in the energy transfer by establishing a lactate shuttle-system. Under this condition, MCT1 favors cellular lactate-uptake, whereas MCT4 rather exports lactate [27]. Thereby, differential MCT1 and MCT4 expression in neighboring cells (slightly and highly glycolytic, respectively) allows the flux of lactate and also other monocarboxylates or ketone bodies from one cell to another. Physiologically, such conditions occur between astrocytes and neurons in the CNS [28] or between fast and slow twitching muscle fibers [29]. In this way, tumor–stroma interactions can be regarded as reminiscent of physiological energy transfer-systems. Accordingly, tumors that utilize a reverse Warburg metabolism are characterized by high MCT1 expression in tumor cells and high MCT4 expression in the surrounding desmoplastic stroma [18,19].

It can be envisioned that, depending on the reciprocal expression of these lactate carriers, metabolic compartmentalization and energy transfer mechanisms are important drivers in the development of clonal variations of cancer cells thereby essentially contributing to the malignant phenotype of a given tumor. This includes the emergence of stem cell-like cancer cells (CSCs) that have a pivotal role in tumor development and progression [30]. Moreover, CSCs are essential for the malignant traits of cancer such as therapy resistance and metastasis. Consequently, the presence of CSCs in their supportive niches created by the tumor microenvironment [31] and their resilience to chemotherapy are regarded as the major cause for disease relapse, as drastically seen in PDAC patients. Thus, understanding the impact of certain metabolic conditions such as the reverse Warburg metabolism in PDAC on the CSC niche is an important issue [32]. The present study therefore investigated how MCT1 driven lactate import as a key process of the reverse Warburg metabolism impacts on the phenotype of PDAC cells and whether stemness properties are particularly favored under this condition.

## 2. Results

### 2.1. Reciprocal MCT1 and MCT4 Expression in PDAC Tissue Reflecting Metabolic Compartmentalization

**Figure 1:**
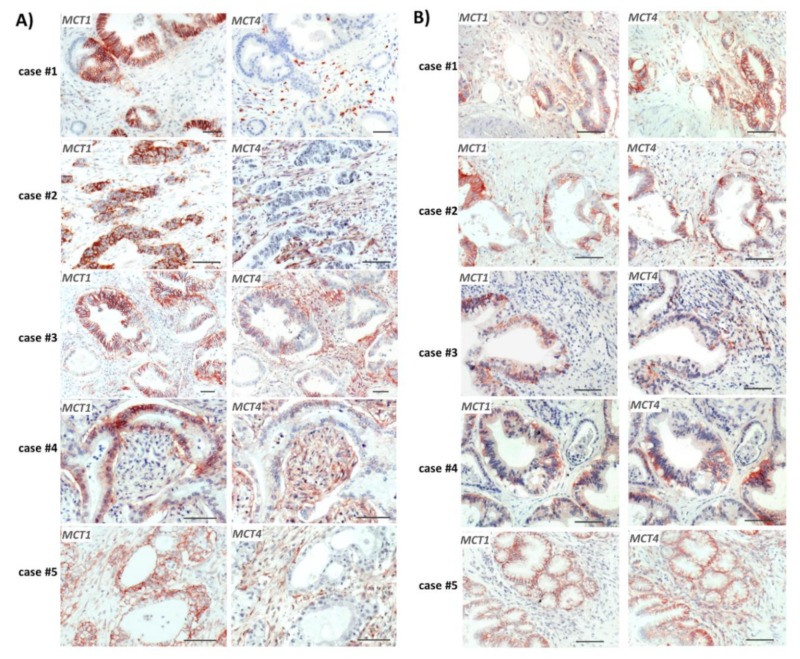
Reciprocal expression of monocarboxylate transporter-1 (MCT1) and -4 (MCT4) in human Pancreatic ductal adenocarcinoma (PDAC) tissues. Using antibodies directed against MCT1 and MCT4, respectively, immunohistochemical analysis was conducted with consecutive formaline-fixed paraffin-embedded (FFPE) tumor sections from PDAC patients (all staged IIb) [33]. Tissue stainings from (**A**) five PDAC cases with MCT1 expression in cancer cells and reciprocal expression of MCT4 largely in stromal cells and (**B**) five PDAC cases with reciprocal MCT1 and MCT4 expression in cancer cells are shown (scale bar = 50 µm).

Consecutive sections of formalin-fixed and paraffin-embedded (FFPE) tumor tissue from PDAC patients (all with re-sectable T3N1M0 tumors) [33] were immunostained with antibodies directed against MCT1 and MCT4, respectively. As shown in Figure 1, considerable expression of MCT1 was seen in several tumor areas. Here, MCT1 was mainly localized at the surface of cancer cells and within dysplastic ductal structures. In these tumoral areas, reciprocal expression of MCT4 predominated in distinct regions. Most of these tissues revealed the strongest MCT4 expression in the tumor stroma that surrounds MCT1 expressing cancer cells (Figure 1A), whereas in other cases (Figure 1B), strong MCT4 expression was detected in cancer cells located in close vicinity to MCT1-expressing PDAC cells within dysplastic ductal structures. Thus, the reciprocal expression pattern of these two lactate carrier proteins indicates metabolic compartmentalization in PDAC tissue.

### 2.2. The Lactate Uptake of PDAC Cells Depends on Their MCT1 Expression

**Figure 2:**
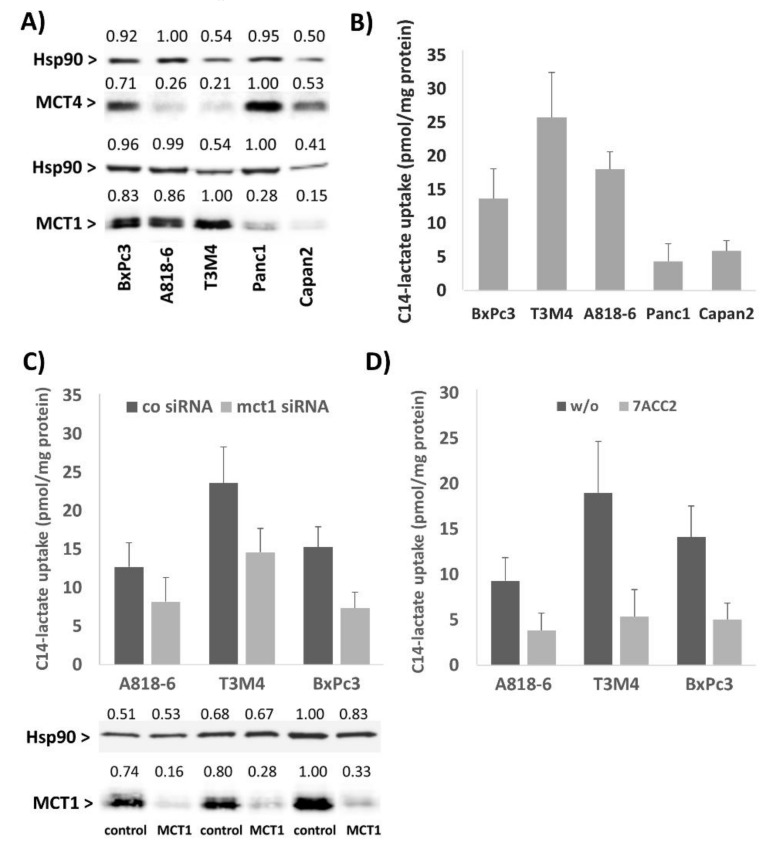
MCT1 expression in PDAC cells and its impact on cellular lactate uptake. (**A**) A818-6, BxPc3, T3M4, Panc1, and Capan2 cells were analyzed by Western blotting for the expression of MCT1 and MCT4. Heat shock protein 90 (Hsp90) expression was determined as loading control. A representative result from three independent experiments is shown. (**B**) A818-6, BxPc3, T3M4, Panc1, and Capan2 cells were submitted to C14-lactate uptake assay. Data express the specific incorporation of C14- lactate normalized to the amount of cellular protein and show the mean ± SD from three inde­pendent experiments. A818-6, BxPc3, and T3M4 cells were (**C**) treated with control or MCT1 siRNA for 48 h or (**D**) either left untreated (w/o) or treated with 20 µM 7ACC2 for 2 h prior to submitting the cells to the C14-lactate uptake assay. Data show the mean ± SD from three independent experiments. The knock-down of MCT1 by siRNA was verified by Western blot shown at the bottom of panel (**C**). Numbers displayed above the images of the Western blots in (**A**,**C**) indicate the relative intensity of each band.

The PDAC cell lines Panc1 and Capan2 with little MCT1 expression and A818-6, BxPc3, and T3M4 expressing MCT1 at high level (Figure 2A) were tested for C14-lactate uptake. As shown in Figure 2A,B, the highest uptake of C14-lactate was seen in T3M4 cells, followed by A818-6 and BxPc3 cells, whereas Panc1 and Capan2 cells took up much less C14-lactate. When knocking down MCT1 expression in A818-6, BxPc3 and T3M4 cells by siRNA (Figure 2C) or when adding 20 µM of the MCT1 inhibitor 7-aminocarboxycoumarin 2 (7ACC2) [26,34] to these cells (Figure 2D), the uptake of C14-lactate was strongly reduced. These data support the view that high MCT1 expression is associated with elevated lactate uptake in PDAC cells.

### 2.3. Treatment of A818-6, T3M4, and BxPc3 Cells with Lactate Protects from Gemcitabine-Induced Apoptosis, an Effect Blocked by the Selective MCT1 Inhibitor 7ACC2

**Figure 3:**
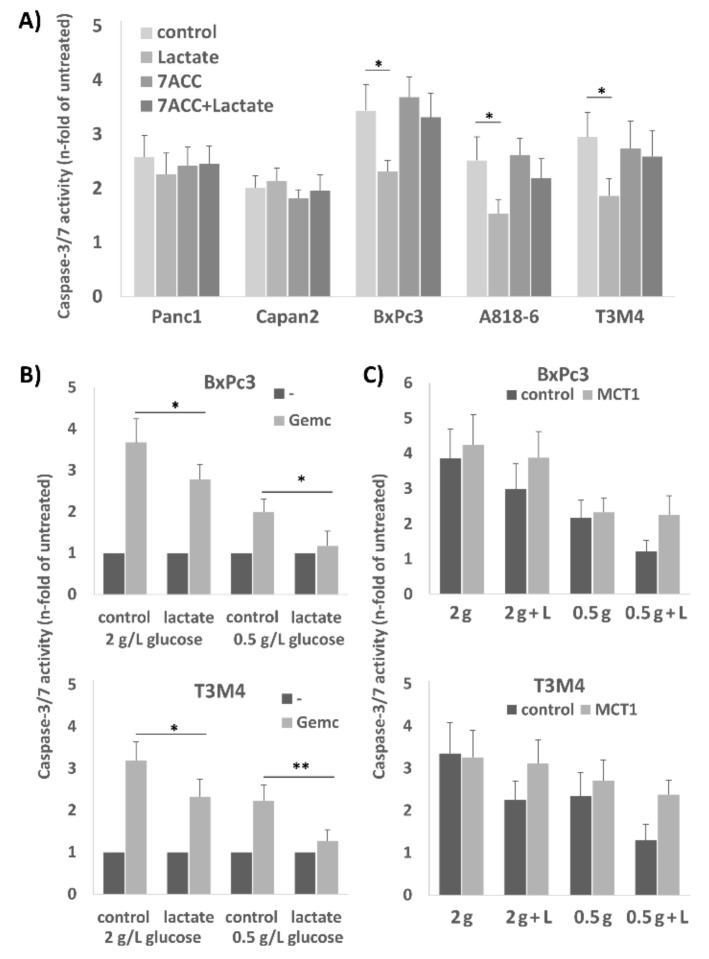
MCT1-driven lactate import protects human PDAC cell lines from anti-cancer drug-induced apoptosis. (**A**) Using the PDAC cell lines A818-6, BxPc3, and T3M4 exhibiting high MCT1 expression or the cell lines Panc1 and Capan2 with low MCT1 expression caspase-3/7 assays were performed to detect apoptosis induction. Cells were either left untreated or were treated with 20 mM lactate for 24 h. Afterwards, cells were left untreated (w/o) or were treated with 20 µg/mL gemcitabine for 40 h. In addition, 20 µM 7ACC2 was added 26 h alone or 2 h before the lactate addition and subsequent gemcitabine treatment. Data are expressed as n-fold caspase-3/7 activity of untreated cells and show the mean ± SD from five independent experiments; * *p* < 0.05. (**B**) BxPc3 and T3M4 cells were cultivated for 72 h in normal medium containing 20 g/L glucose or in low glucose medium with 0.5 g/L glucose either in the absence or presence of 20 mM lactate. Then, gemcitabine induced apoptosis was analyzed after 40 h by caspase-3/7 assay. Data are expressed as n-fold caspase-3/7 activity of untreated cells and show the mean ± SD from five independent experiments; * *p* < 0.05, ** *p* < 0.01 when comparing lactate treated versus untreated cells. (**C**) BxPc3 and T3M4 cells were pretreated with control or MCT1 siRNA for 24 h and then cultivated in normal medium (2 g) or low glucose medium (0.5 g) for 72 h either without or with lactate. Then, gemcitabine induced apoptosis was analyzed after 40 h by caspase-3/7 assay. Data are expressed as n-fold caspase-3/7 activity of untreated cells and show the mean ± SD from three independent experiments.

Next, we investigated the impact of lactate on the chemosensitivity of PDAC cells. As shown by caspase-3/7 assay, gemcitabine-induced cell death of the MCT1 expressing cell lines A818-6, BxPc3, and T3M4 was reduced upon preincubation with 20 mM lactate (Figure 3A). By contrast, Capan2 and Panc1 cells which express MCT1 at rather low level did not show an alteration of gemcitabine-induced apoptosis rates when receiving lactate pretreatment (Figure 3A).

After treatment with 20 µM 7ACC2, the gemcitabine-induced apoptosis rate was increased in A818-6, BxPc3, and T3M4 cells, and the rescuing effect by lactate treatment was abolished (Figure 3A). In Panc1 and Capan2 cells, the addition of 7ACC2 did not affect gemcitabine-induced apoptosis.

Since the shuttling of lactate in reverse Warburg cells is more effective under glucose restriction, BxPc3 and T3M4 cells were next cultured at normal (2 g/L) or low (0.5 g/L) glucose concentration for 72 h followed by gemcitabine treatment (Figure 3B). Under low glucose conditions, both cell lines showed already a lower sensitivity against gemcitabine induced apoptosis (1.99 and 2.23 fold of untreated compared to 3.67 and 3.19 fold of untreated, respectively). Administration of 20 mM lactate led to an even more pronounced chemoresistance under glucose shortage (1.17 and 1.26 fold of untreated, respectively) as compared to normal glucose supply (2.78 and 2.33 fold of untreated, respectively). The knock-down of MCT1 by siRNA pretreatment (Figure 3C) abolished the effect of lactate on gemcitabine-induced apoptosis in T3M4 and BxPc3 cells. This was seen under both culture conditions in normal and low glucose medium.

### 2.4. Clonal Growth of BxPc3 and T3M4 Cells under Glucose Shortage is Increased by Lactate: An Effect Blocked by 7ACC2

**Figure 4:**
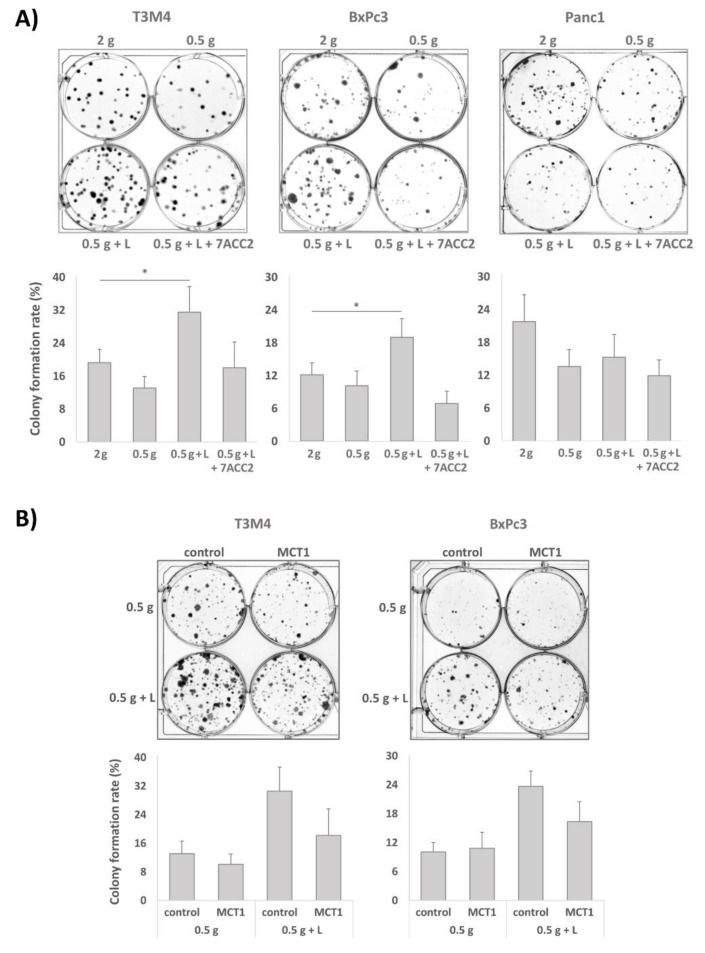

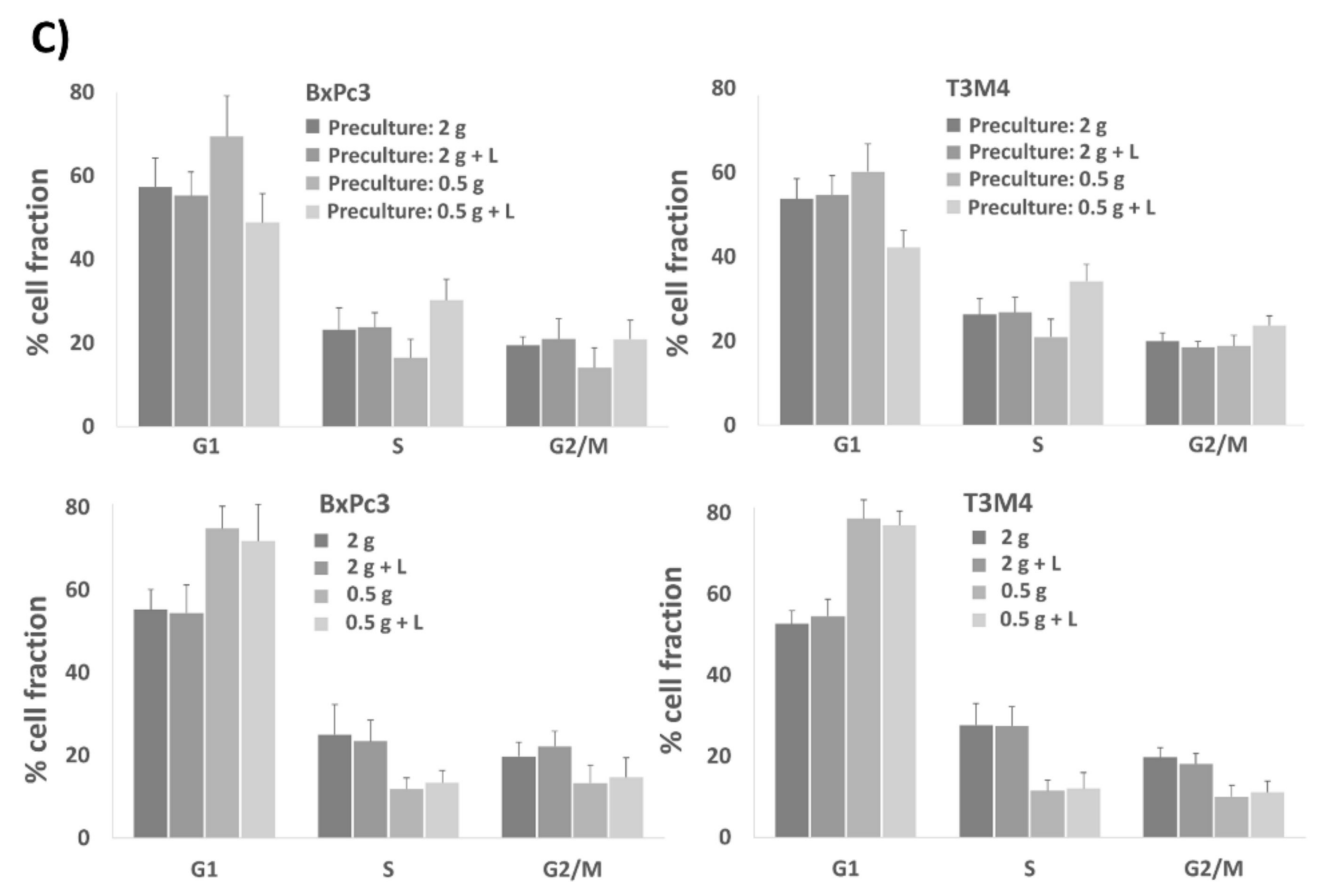
Lactate treatment augments colony formation capacities of T3M4 and BxPc3 cells under glucose restriction. (**A**) BxPc3, T3M4, and Panc1 cells were precultured in normal medium (2 g) or under glucose restriction using medium with 0.5 g/L glucose (0.5 g) for 72 h, either in the absence or presence of 20 mM lactate (0.5 g + L and 2 g + L, respectively) and additionally of 20 µM 7ACC2. Then, the cells were reseeded at a density of 200 cells/well on six-well plates in normal culture medium. After 1 week, medium was discharged, and cells were washed with PBS followed by staining with crystal violet for visualizing colonies. Representative images are depicted, and data shown below represent the mean value ± SD from five independent experiments; * *p* < 0.05. (**B**) BxPc3 and T3M4 cells were treated with control or MCT1 siRNA for 24 h and then precultured under glucose restriction in the absence or presence of 20 mM lactate (0.5 g and 0.5 g + L, respectively) for 72 h. Afterwards, cells were reseeded in normal medium and colony formation was measured as described above. Representative images are depicted, and data shown below represent the mean value ± SD from three independent experiments. (**C**) Deriving from 72 h preculture in the normal medium ± lactate (2 g, 2 g + L) or low glucose medium ± lactate (0.5 g, 0.5 g + L), BxPc3 and T3M4 cells were reseeded in normal medium for 48 h and then submitted to propidium iodide (PI)-cell cycle analysis (upper panel), or submitted to PI-cell cycle analysis prior to reseeding (lower panel). The mean values ± SD from six independent experiments are shown (see also Table 1).

Next, the impact of lactate under normal or reduced glucose concentrations on colony formation was analyzed in the MCT1 expressing PDAC cell lines BxPc3 and T3M4. These cells were first cul­tured in normal medium containing 2 g/L of glucose or with low glucose (0.5 g/L) medium supple­mented with 20 mM lactate or not, either in the absence or presence of 10 µM 7ACC2. Then, after 3–4 days cells were trypsinated and reseeded at low density in normal medium again. After 6–10 days, colony formation was evaluated. Interestingly, both cell lines showed the greatest colony formation rate within 6-10 days when precultured in low glucose medium supplemented with lactate (Figure 4A). This colony formation rate exceeded even the rate of cells preincubated in normal medium and was not seen in the absence of lactate and, most notably, when the MCT1 inhibitor 7ACC2 was added in advance. No effect of lactate addition under glucose restriction on the colony formation rate was seen in Panc1 cells (Figure 4A) expressing MCT1 at low level (see above and Figure 2A).

The MCT1 dependency in BxPc3 and T3M4 cells was confirmed by siRNA treatment which was conducted prior to the 72 h preculture. As seen in Figure 4B, MCT1 knockdown diminished the effect of the lactate treatment under glucose restriction on colony formation of both cell lines after their re-exposure to the glucose medium. Thus, under glucose restriction, the MCT1-driven import of lactate results in the enrichment of PDAC cells for a phenotype with higher self-renewal capacity, thereby leading to greater colony formation rates upon re-exposure to glucose.

### 2.5. The Presence of Lactate during Preculture under Glucose Restriction Primes BxPc3 and T3M4 Cells for Accelerated Cell Cycle Progression upon Reseeding in Normal Medium

Next, we studied the effect of lactate on cell proliferation. Propidium iodide (PI) cell cycle analysis (Figure 4C upper panel and Table 1A) revealed that, compared to cells subject to preculture in normal medium (G1: 57.4% and 53.7%, respectively) or low glucose medium without lactate (G1: 69.4% and 60.2%, respectively), the G1-fraction of BxPc3 and T3M4 reseeded in normal medium for 2 days was reduced (G1: 48.8% and 42.2%, respectively) if these cells had been precultured for 72 h in low glucose (0.5 g/L) medium plus 20 mM lactate. Accordingly, the content of cells in the S-phase (30.3% and 34.2%, respectively) and G2/M phase (20.9% and 23.6%, respectively) was greater in BxPc3 and T3M4 cells precultured in low glucose plus lactate medium than in those cells precultured in normal medium (S: 23.2% and 26.3%, respectively, G2/M: 19.5% and 19.9%, respectively) or low glucose medium without lactate which resulted in a significant delay in cell cycle progression (S: 16.5% and 20.9%, respectively, G2/M: 14.1% and 18.9%, respectively). In contrast to these effects after reseeding the cells in normal medium, PI cell cycle analysis (Figure 4C, lower panel, and Table 1B) of the cells prior to reseeding revealed that the G1-fraction of BxPc3 and T3M4 cells cultured for 72 h in low glucose medium (0.5 g/L) was greatly increased (G1: 74.9% and 78.6%, respectively) compared to cells cultured in normal medium (G1: 55.3% and 52.6%, respectively), and the addition of lactate only slightly altered the effect of glucose restriction (G1: 71.9% and 76.9%, respectively). Accordingly, the decreased content of both cell lines in the S- and G2/M-phase was lower under glucose restriction without an effect by lactate. Overall, these data indicate that lactate does not directly affect the cell cycle progression in glucose-starved BxPc3 and T3M4 cells but primes them for an accelerated cell cycle upon re-exposure to glucose.

### 2.6. BxPc3 and T3M4 Cells Reseeded in Normal Medium Exhibit Greater Drug Resistance When Precultured with Lactate under Glucose Restriction

**Figure 5:**
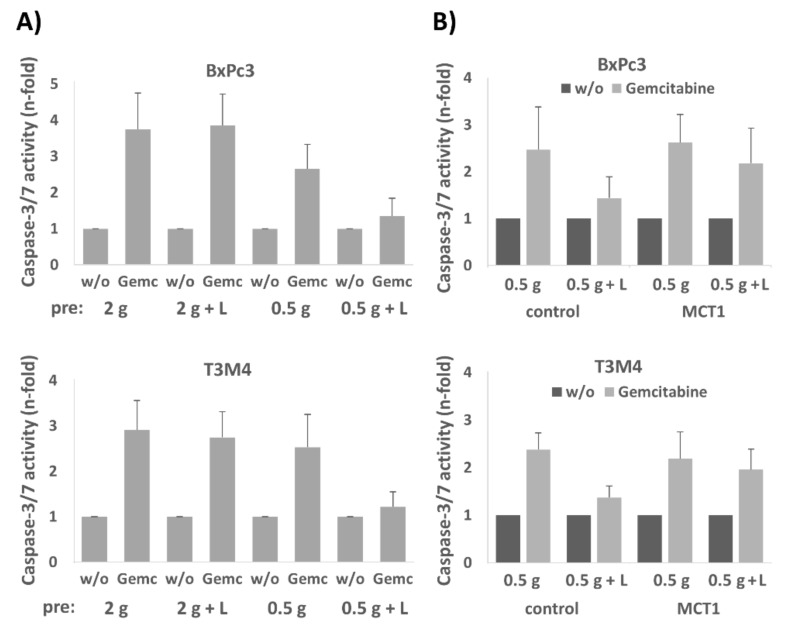
The presence of lactate during preculture under glucose restriction favours drug resistance of BxPc3 and T3M4 cells reseeded in normal medium. Deriving from 72 h preculture in the normal medium ± lactate (2 g, 2 g + L) or low glucose medium ± lactate (0.5 g, 0.5 g + L), BxPc3 and T3M4 cells, either (**A**) without or (**B**) with control and MCT1 siRNA pretreatment, were reseeded in normal medium for 24 h and were then left untreated or treated with 20 µg/mL gemcitabine for 40 h. Gemcitabine induced apoptosis was analyzed by caspase-3/7 assay. Data are expressed as n-fold caspase-3/-7 activity and show the mean values ± SD from three independent experiments.

In order to investigate how the presence of lactate under glucose restriction impacts drug resistance after glucose re-exposure, BxPc3 and T3M4 cells were differentially precultured and reseeded in normal medium, as described above. As shown in Figure 5A, BxPc3 and T3M4 reseeded from preculture under glucose restriction and lactate supplementation exhibited only marginal apoptotic responses after treatment with gemcitabine (1.35 and 1.22 fold of untreated, respectively) when compared with those cells derived from normal medium precultures (3.65 and 2.91 fold of untreated, respectively). Cells reseeded from low glucose preculture without supplementation of lactate exhibited moderately reduced apoptotic responses (2.66 and 2.53 fold of untreated, respectively). The knockdown of MCT1 expression in these two cell lines abrogated the resistance inducing effect of the preculture with lactate under glucose restriction. As shown in Figure 5B, BxPc3 and T3M4 cells transfected with MCT1 siRNA exhibited greater apoptotic responses to gemcitabine (2.18 and 1.96 fold of untreated, respectively) as compared to control siRNA transfected cells (1.44 and 1.37 fold of untreated, respectively). These data indicate that lactate primes glucose-starved PDAC cells for a chemoresistant phenotype after their re-exposure to glucose.

### 2.7. Effect of Lactate Exposure under Glucose Restriction on Stemness Marker and Reprogramming Factor Expression in BxPc3 and T3M4 Cells

**Figure 6:**
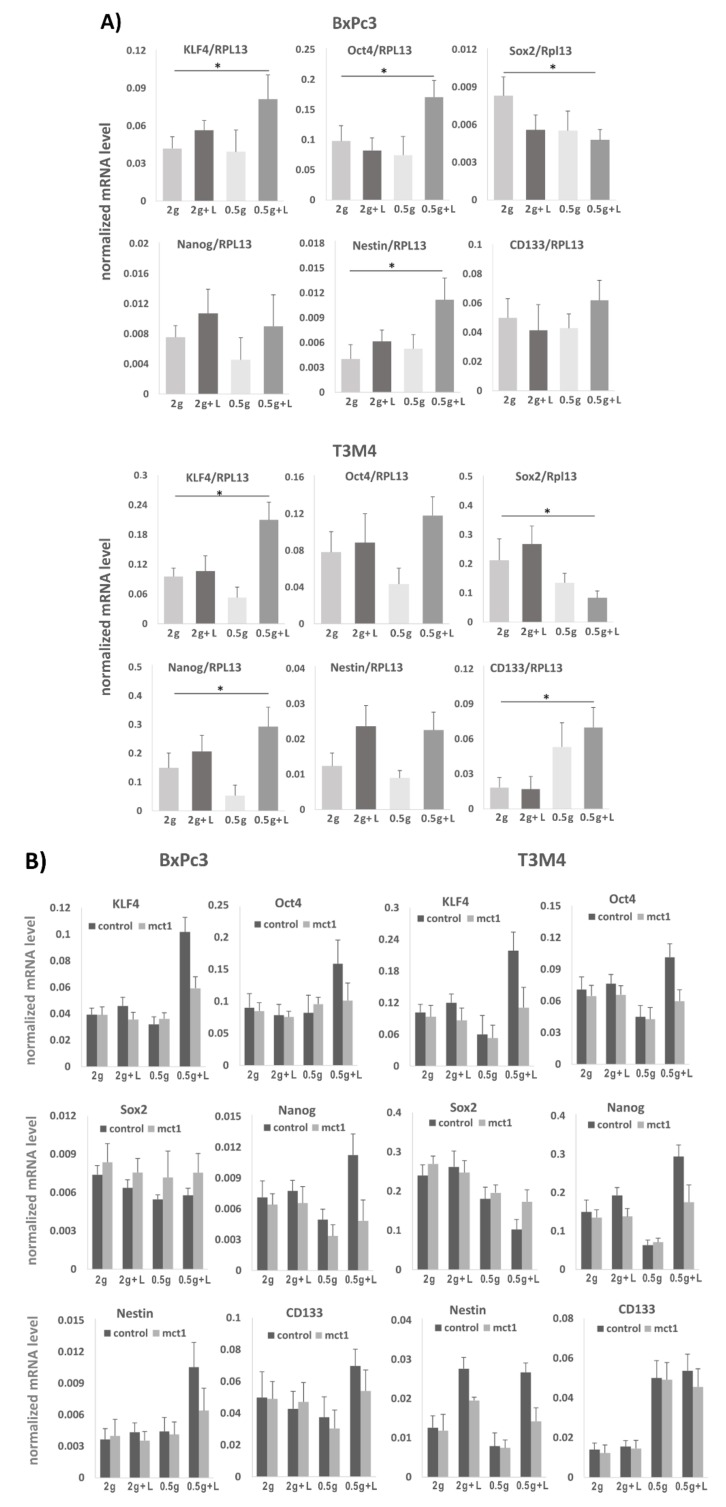

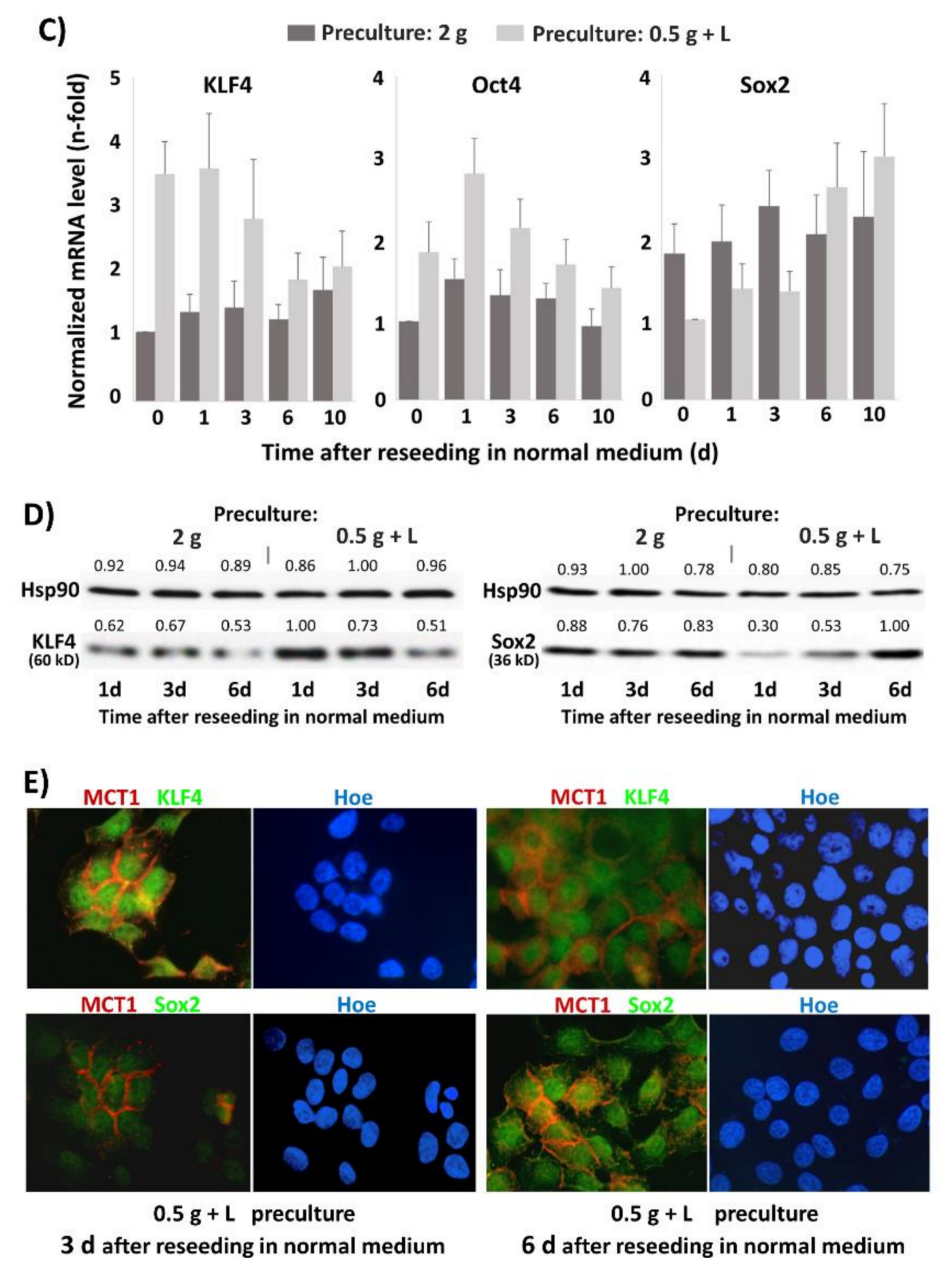
Lactate treatment increases stemness marker and reprogramming factor expression in T3M4 and BxPc3 cells under glucose restriction. (**A**) BxPc3 and T3M4 cells were precultured for 72 h under glucose restriction (0.5 g/L) or regular glucose supply (2 g/L) either in the presence or absence of 20 mM lactate and subsequently grown in normal medium for 24 h. (**B**) BxPc3 and T3M4 cells were treated with control or MCT1 siRNA and then differentially precultured/cultured as described above. RNA was collected and used for qPCR analysis of stemness markers Nestin and CD133 as well as the reprogramming factors Nanog, Sox2, KLF4, and Oct4. RPL13 served as a housekeeper. (**C**) T3M4 cells were precultured for 72 h in normal medium (2 g) or low glucose medium with 20 mM lactate (0.5 g + L), followed by reseeding in normal medium. RNA samples taken right before reseeding or 1, 3, 6 and 10 days later were analyzed by qPCR for Sox2, Oct4 and KLF4 expression (RPL13 served as house­keeping gene). All qPCR derived data represent the mean value ± SD from four (A) and three (B & C) independent experiments; * *p* < 0.05. (**D**) T3M4 cells were precultured for 72 h in normal medium (2 g) or low glucose medium with 20 mM lactate (0.5 g + L), followed by reseeding in normal medium for 1, 3, and 6 days. Then, KLF4 and Sox2 expression was analyzed by Western blotting (Hsp90 was used as loading control). A representative result from three independent experiments is shown. Num­bers displayed above the images indicate the relative intensity of each band. (**E**) T3M4 cells from 72 h preculture with low glucose medium in the presence of lactate (0.5 g + L) were reseeded on cover slips with normal medium for 3 and 6 days and then analyzed by immunofluorescence microscopy for KLF4, Sox2, and MCT1 expression. A representative result from three independent experiments is shown.

Since lactate promotes self-renewal abilities along with a drug resistant phenotype, it was next analyzed whether these alterations go along with an altered expression of stemness markers and reprogramming factors. For this purpose, BxPc3 and T3M4 cells cultured in normal or low glucose medium, either with lactate (20 mM) or without, were analyzed for stemness marker and reprogramming factor expression by qPCR after reseeding and culturing the cells in normal medium. As shown in Figure 6A, Nestin, KLF4 and Oct4 were most significantly elevated in BxPc3 cells grown after lactate pretreatment under glucose restriction and reseeding in normal medium, whereas Sox2 was decreased. In T3M4 cells, preconditioning with lactate pretreatment under glucose resitriction resulted in the most significant upregulation of Nanog and, again, of KLF4 and Oct4, whereas Sox2 was downregulated (Figure 6A). 

The MCT1 dependency of these lactate mediated effects on stemness marker and reprogramming factor expression was validated by transfecting BxPc3 and T3M4 cells with MCT1 siRNA prior to the differential precultures. In comparison with control siRNA treatment, both cell lines subjected of MCT1 siRNA treatment showed a decreased effect of lactate pretreatment —particularly under glucose restriction - on KLF4, Nestin, Sox2, Oct4 and Nanog expression (Figure 6B).

Interestingly, the effect of lactate pretreatment on the expression of KLF4, Oct4, and Sox2 in T3M4 cells lasted for over 72 h after re-exposure to normal medium (Figure 6c). While the expression of KLF4 and Oct4 declined thereafter, the expression of Sox2 increased until days 6–10. Thus, there seems to be a reciprocal effect of lactate pretreatment on KLF4 and Oct4 expression on the one hand and Sox2 expression on the other hand, that was reversed after a certain time of glucose re-exposure. These effects could be also seen by Western blot analysis (Figure 6d) which revealed an enhanced KLF4 but lower Sox2 expression in T3M4 cells at days 1 and 3 after reseeding the cells pretreated with low glucose plus lactate. By contrast, Sox2 expression increased in both cell lines within 3-6 days after reseeding. Immunofluorescence staining of T3M4 cells (Figure 6e) confirmed high KLF4 expressing and rather Sox2 negative clones at early periods (1-3 days) and clones with higher Sox2 expression at later periods (6 days) when KLF4 was less expressed. This observation is intriguing, since a high ratio of KLF4/Sox2 and Oct4/Sox2 ratio has been reported to be associated with greater self-renewal capacity [35].

### 2.8. Areas with Pronounced MCT1 Expression Colocalize with KLF4 in PDAC Tissues

**Figure 7:**
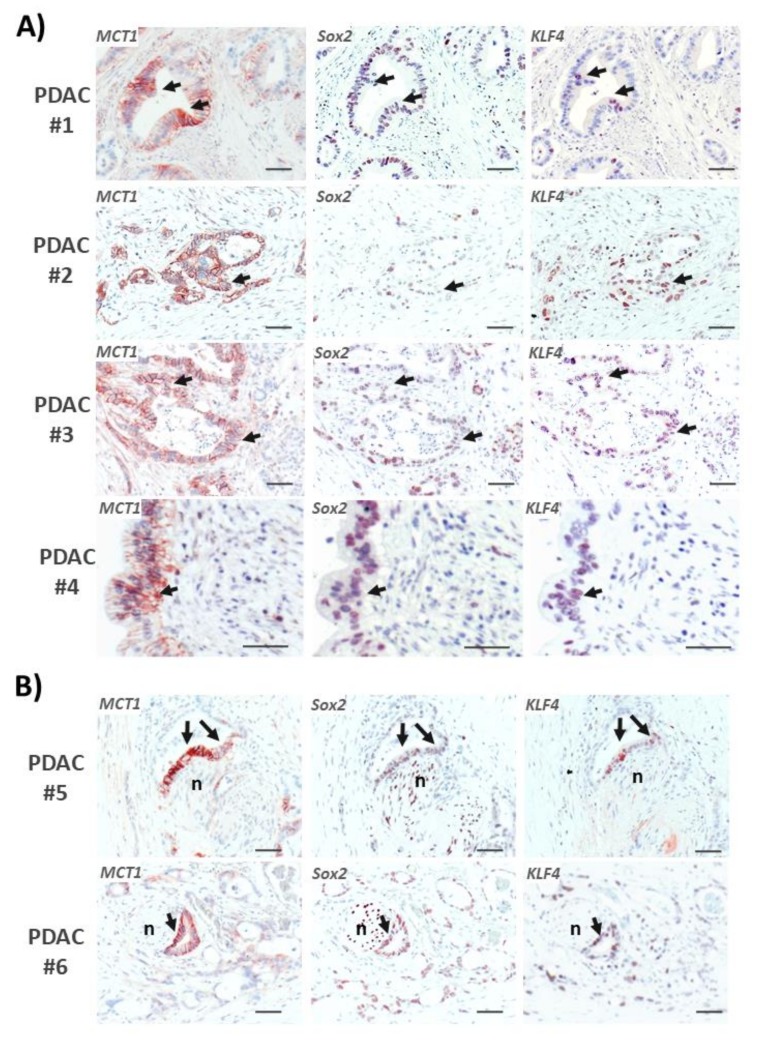
MCT1 expressing PDAC tissue areas colocalized with KLF4, partially reciprocally to Sox2. Consecutive sections of FFPE tumor tissues from PDAC patients were stained with antibodies directed against MCT1, KLF4, and Sox2, receptively. Images from (**A**) four PDAC tissues and (**B**) from two perineural invasive PDACs (PNI) are shown. Arrows indicate MCT1 positive cells exhibiting colocalization with strong KLF4 and weak Sox2 expression; n: neural cells. (scale bar = 50 µm).

In order to verify the association of MCT1 expression and stemness properties in human PDAC tissue, consecutive sections of FFPE tumor tissues from PDAC patients (all with resectable T3N1M0 tumors) [33] were immunostained with antibodies directed against MCT1, KLF4, and Sox2, respectively. As shown in Figure 7A, those PDAC areas revealing strong staining of MCT1 partially exhibited low or even no expression of Sox2 but considerable KLF4 expression. By comparison, Sox2 expression was also detectable in PDAC cells exhibiting strong MCT1 staining, but lacking KLF4 expression, or in PDAC cells with low or even no MCT1 expression. As another interesting observation, we detected in some cases strong MCT1 and KLF4 co-expression in areas of perineural invasion (PNI) of PDAC cells which were to some extent less positive or even negative for Sox2 staining. In these PNI regions, Sox2 was more strongly expressed by the nerve cells (Figure 7B).

## 3. Discussion

Depending on distinct metabolic conditions, such as a high rate of aerobic glycolysis or of oxidative phosphorylation (oxPhos), tumor cells either need to get rid of lactate or in turn may use it as substrate for their energy metabolism [14]. The flux of metabolites such as lactate, and thereby of energy, released by one cell type (e.g., glycolytic stromal fibroblast and/or cancer cells) and consumed by another one (e.g., oxPhos cancer cell) greatly adds to the tumoral ecosystem and ensures individual cellular fitness [15,16]. It can be envisioned that each tumor cell, residing in a complex ecosystem composed of heterogeneous populations of neighboring tumor cells and stromal cells (e.g., fibroblasts, immune cells), compete for nutrients like glucose and oxygen on the one hand, but mutually provide each other with certain metabolites on the other hand [36,37,38]. Together with sporadic genetic alterations these metabolic restraints at the primary tumor site strongly shape the phenotypic heterogeneity [39] and may favor the emergence of single cancer cells exhibiting stemness-like phenotypes. This scenario relates to the emergence of certain oncometabolites that drive alterations in the epigenome associated with the acquisition of stem cell properties [40]. Hence, it was recently shown that metabolites such as 2-hydroxyglutarate, ketone bodies or lactate exert a strong impact on epigenomic editors thereby affecting the reprogramming barrier in cancer cells [40].

Representing important key molecules of such metabolite flux conditions, the lactate carrier proteins MCT4 and MCT1 are essential for the manifestation of a highly glycolytic/lactate releasing phenotype on the one side and the capability to take advantage of consuming lactate or other metabolic substrates on the other side. Our present study revealed that the MCT1-driven lactate import in PDAC cells provides considerable protection from apoptotic cell death in response to anti-cancer drug treatment. Accordingly, knock-down of MCT1 or application of the specific MCT1-lactate import inhibitor decreased the chemoresistance of these PDAC cells.

In line with the fact that refractoriness to chemotherapy is a hallmark associated with cancer stemness [41,42,43], we could further show that MCT1-driven lactate uptake favors stemness properties. Thus, under glucose restriction and exposure to lactate, PDAC cells are characterized by a greater capacity of colony formation that is seen after their re-exposure to normal glucose supply. This obvious priming effect by the reverse Warburg condition (low glucose/high lactate) along with the MCT1 driven lactate import is accompanied by the altered expression of stemness factors, including a transient increase of KLF4 expression, and also increased expression of Oct4, Nestin, and Nanog. In a reciprocal fashion, the reverse Warburg condition decreased Sox2 expression depending on MCT1-dependent lactate shuttling, whereas the re-exposure to glucose increased Sox2 but decreased KLF4 expression. Thus, the high KLF4/Sox2 ratio after treatment under reverse Warburg conditions and still seen at the beginning of glucose re-exposure turned to a low KLF4/Sox2 ratio during the glucose induced propagation/transit-amplifying phase. In a previous study [35], it was shown that those subpopulations of cancer cells exhibiting high expression of KLF4 and/or Oct4 and low expression of Sox2 produced much more colonies than subpopulations with high Sox2 expression and lower KLF4 and/or Oct4 expression. The authors concluded from these data that KLF4/Oct4 are specially required for high self-renewal capacity and giving rise to Sox2 expressing cells in their progeny. More recently, a core function of KLF4 for reprogramming in concert with Sox2 and independence of Oct4 was demonstrated [44].

Meanwhile, several studies have confirmed the fundamental role of the sequential expression of these three reprogramming factors [45,46,47,48]. It can be speculated that the reverse Warburg compartment harbors a subpopulation of cancer cells that are characterized by a quiescent stem cell-like phenotype. These cells show a high expression of KLF4/Oct4 and a rather low expression of Sox2 and are maintained by the import of lactate under glucose restriction. Upon re-exposure to glucose, these cells are recruited into the induced and further into the transit-amplifying phase, giving rise to rapidly proliferating cell clones. This is accompanied by a switch to the Warburg metabotype and an increase of the Sox2/KLF4-Oct4 ratio. In support of these findings, previous work already indicated that the reverse Warburg metabolism favors a cellular phenotype characterized by lower mitotic activity but full self-renewal potential and it was reported that lactate, pyruvate and ketone bodies can drive cancer cells into a stem cell-like phenotype [49,50,51,52]. Physiologically, such a condition reflects a starvation period when cells need to utilize alternative catabolic fuels unless glucose becomes sufficiently available again. Then, these reverse Warburg cancer cells are particularly recruited into the transit-amplifying state, characterized by a metabolic shift towards high rate glycolysis (Warburg) and high mitotic activity. Again, few cells remain in the reverse Warburg metabolism and persist in the stem cell-like state favored by lactate utilization [53]. The role of lactate in inducing CRC characteristics under glucose restriction could relate also to an altered lipid metabolism that was recently linked with cancer stemness, too [54]. Thus, the lactate uptake may act in concert with changes in the rate of fatty acid synthesis and transport when glucose supply is low. Alternatively, fatty acid metabolism, including fatty acid oxidation, could add to the stemness inducing effect of lactate under glucose starvation [55].

Thus, reverse Warburg cancer cells may have particular therapeutic relevance. Given that most conventional cancer therapies target the more proliferative cells in a tumor which execute the glycolytic Warburg metabolism, the mass of glucose consuming and lactate releasing tissue is eradicated. Amongst the few remaining cells after therapy, less proliferative reverse Warburg cells may have survived—partially adopting a stem cell phenotype. Through the elevated supply with glucose upon eradication of the Warburg cell mass, these cancer stem cell-like cells may initiate rapid growth and expansion of new cancer cell clones giving rise to recurrent disease and metastasis.

Based on the role of the reverse Warburg metabolism as a condition favoring cancer cell stemness, MCT1 may represent a stemness marker, as suggested previously [56]. Moreover, the efficient blockade of MCT1-driven lactate import, e.g., by 7ACC2, in PDAC cells and the inhibitory effects on stemness properties underscores the potential of targeting reverse Warburg cells in cancer therapy. Similar observations have been made with glioblastoma stem cells that are maintained by MCT1 driven lactate transport [57]. Thus, concepts have been already discussed that consider disrupting the metabolic coupling between cancer and stroma cells as pivotal in breaking resistance to conventional therapies [57,58,59,60]. Such a treatment might have great potential in a stroma rich tumor such as PDAC, suppressing particularly stem cell-like and dormant tumor cell clones that drive recurrence and metastasis formation [61]. However, the environmental context in which MCT1 exerts its effects on tumor growth seems to be an important issue. A recent report showed a correlation between MCT1 expression and better prognosis in PDAC patients [62]. Thus, it is conceivable that the MCT1 expression in extended areas of the tumor is attributable to moderately glycolytic tumor cells of the Warburg phenotype where MCT1 can act as exporter rather than as importer of lactate [27]. By contrast, when facing highly glycolytic tumor or stroma cells that secrete excessive amounts of lactate through MCT4, as seen under conditions of metabolic compartmentalization, some MCT1 expressing tumor cells switch to lactate importing (reverse Warburg) cells. Depending on this particular microenvironment, MCT1-expressing cells include those adopting CSC characteristics and accounting for the poor prognosis associated with metabolic compartmentalization and the reverse Warburg metabolism.

## 4. Materials and Methods

### 4.1. Cell Lines and Culture

The human PDAC cell lines Panc1 and BxPc3 were from the DSZM (Braunschweig, Germany). T3M4 cells were kindly provided by H. Friess (Heidelberg, Germany) and A818-6 cells were a gift from H. Kalthoff (Kiel, Germany). Culture conditions were as described recently [63,64]. Cell line authenticity was checked by STR-profiling.

### 4.2. RNA Preparation and Real-Time PCR

Isolation of RNA, reverse-transcription into single-stranded cDNA and real-time PCR (*PikoReal*-System, Thermo-Fisher-Scientific, Schwerte, Germany) using the SYBR-Green assay (Thermo-Fisher-Scientific) were carried out as described [65]. All primers (Eurofins, Ebersberg, Germany) were used at a final concentration of 0.2 µM. Cycling conditions were: 95 °C, 7 min initial denaturation, followed by 45 cycles at 95 °C, 5 s/60 °C, 30 s. The following primer sets were used: KLF4 forw/rev; 5′-GGGAG AAGACACTGCGTCAA-3′/5′-GGAAGTCGCTTCATGTGGGA-3′,Oct4 forw/rev; 5′-GGTGGAGGA AGCTGACAACA-3′/5′-GTTCGCTTTCTCTTTCGGGC-3′,Sox2 forw/rev; 5′-TCCCATCACCCACAG CAAATGA-3′/5′-TTTCTTGTCGGCATCGCGGTTT-3′, Nanog forw/rev; 5′-ACATGCAACCTGAAG ACGTGTG-3′/5′CATGGAAACCAGAACACGTGG-3′, Nestin forw/rev; 5′-CACGTACAGGACCCT CCTGGA-3′/5′TCCTAGGGAATTGCAGCTCCAG-3′, CD133 forw/rev; 5′-ATGCTCTCAGCTCTCC CGC-3′/5′TTCTGTCTGAGGCTGGCTTG-3′, RPL13 forw/rev; 5′-CCTGGAGGAGAAGAGGAAAG AGA-3′/5′TTGAGGACCTCTGTGTATTTGTCAA-3′.

### 4.3. Western Blotting

Total cell-lysates were prepared, separated by SDS-PAGE and submitted to Western blotting as described before [26,64]. Blots were analyzed with the ChemiDoc gel documentation system (Biorad, Munich, Germany). Relative band intensities were calculated by the QuantityOne software (Biorad,), and the strongest band intensity was set to 1.00. All original western blot figures can be found in the Supplementary Materials file.

### 4.4. siRNA Transfection

For siRNA (Qiagen, Hilden, Germany) transfection, cells grown in 12 well plates were transfected using 6 µL of *HiPerfect*-reagent (Qiagen) with 150 ng/well of control-siRNA (Qiagen) or MCT1-siRNA (no.SI03246614, Qiagen).

### 4.5. Colony Formation Assay

The BxPc3 and T3M4 cells were precultured in normal (2 g/L) or reduced (0.5 g/L) glucose medium either in the presence or absence of 20 mM lactate or 20 µM 7ACC2. After 72 h, cells were collected and seeded in 6 well plates (200 cells/well). Culture continued with normal medium (2 g/L glucose) for 7–10 days. Then, cells were washed twice with PBS, fixed with methanol/acetic acid (3:1) for 5 min, and stained with 0.1% (*w*/*v*) crystal violet. Plates were photographed using the Chemidoc-XRS^TM^transiluminator (BioRad). Colonies > 0.25 mm diameter were counted, and plating efficiency was calculated as ratio of colony number/cells initially seeded.

### 4.6. Lactate Uptake Assay

Lactate uptake by PDAC cells was examined using uniformly labelled 14C-lactate (Hartmann Analytics, Braunschweig, Germany) and following a protocol described recently [26].

### 4.7. Propidium Iodide Staining

After trypsinization, cells were washed twice in cold PBS containing 5 mM EDTA (PBSE) and then resuspended in 500 µL PBSE. For fixation, 500 µL chilled EtOH was added dropwise and the mixture was incubated at room temperature for 30 min. Fixed cells were collected by centrifugation, resuspended in 500 µL PBSE, incubated with 20 µg RNaseA for 30 min at room temperature and subsequently stained with propidium iodide (PI) by adding 500 µL of a 200 mg/mL PI-stock solution. Samples were stored at 4 °C in the dark until counting using a FACSVerse cytometer (Becton Dickinson, New Jersey, USA.).

### 4.8. Measurement of Caspase-3/7 Activity

Caspase-3/7 activity was measured making use of the Caspase-Glo^®^ assay (Promega, Mannheim, Germany) according to the manufacturer’s instructions and as described [65]. Samples were measured in duplicates and resulting values were normalized to the respective protein concentration.

### 4.9. Immunofluorescence Microscopy

The T3M4 cells from preculture under glucose restriction in the presence of lactate were reseeded on coverslips at a cell number of 500 and further cultured in regular medium for 3 or 6 days. Then, cells were fixed with methanol/acetone (1:1), washed with PBS, blocked with 4% BSA/PBS and incubated with Sox2 (rabbit, 2748S, Cell Signaling, Frankfurt, Germany; 1:100) or KLF4 (rabbit 12173S, Cell Signaling; 1:100) primary antibodies together with a MCT1 antibody (mouse, GT14612, Sigma, St. Louis, MO, USA.; 1:200) overnight in 4% BSA/PBS. Then, after extensive washing in PBS, AlexaF488 (anti-mouse) and AlexaF546 (anti-rabbit) conjugated secondary goat antibodies (Thermo Fisher, Schwerte, Germany) were added at 1:200 dilution in 4% BSA/PBS for 1 h at room temperature in the dark, together with Hoe35564 nuclear staining dye. After washing in PBS, cover slips were fixed with Fluorsave Reagent (Calbiochem, Darmstadt, Germany) and analyzed with an Axioplan 2 microscope (Zeiss, Jena, Germany).

### 4.10. Patients and Tissues

Pancreatic tissues were obtained from patients during surgery. Conservation of PDAC tissues and histopathological diagnosis were performed at the Institute of Pathology, UKSH Campus Kiel. Only PDAC patients with a tumor disease pathologically staged II b (T3, tumor size > 4 cm; N1, spread to ≤ 3 lymph nodes; M0, no spread to distant sites) were included in the study [33].

### 4.11. Immunohistochemistry

Consecutive 3 µm sections of FFPE tumor tissues from twenty-one PDAC patients were used. Deparaffinization of tissue sections was performed by incubating sections two times in xylene for 10 min. Afterwards, samples were rehydrated applying a descending alcohol series, simultaneously blocking endogenous peroxidases by adding 1.5% (*v*/*v*) H_2_O_2_. Then, tissue sections were washed for 10 min with PBS before antigen retrieval was performed by incubating the sections in a microwave oven for 20 min in pre-warmed antigen retrieval buffer (citrate buffer, pH 6.0). Unspecific binding-sites were blocked by incubation in PBS supplemented with 0.3% (*v*/*v*) Triton X-100 (PBS-T) and 4% (*w*/*v*) BSA for 1 h at RT. Immunostaining was carried out overnight at 4 °C using MCT1 and MCT4 (mouse, F10, Santa Cruz, Heidelberg, Germany) antibodies at 1:150 dilutions in 1% BSA and 0.3% Triton-X in PBS. KLF4 and Sox2 antibodies were incubated at 1:50 and 1:100 dilution, respectively. Immunostaining was visualized using the EnVision + system-HRP labelled polymer anti-mouse or anti-rabbit, followed by administration of AEC Substrate (both Dako Diagnostika, Hamburg, Germany). Mayer’s Haemalaun served as counterstain (AppliChem, Darmstadt, Germany). Respective isotype controls were used to verify staining specificity, revealing no or only weak staining. Finally, immunostainings were evaluated using an Axioplan 2 microscope (Zeiss, Jena, Germany).

### 4.12. Statistical Analysis

As indicated in the figure legends, normally distributed data were evaluated by two-tailed Student’s *t*-test (using Excel 2013 Software run on Microsoft Windows 8.1, Redmond, WA, U.S.A.) assuming equal variance (*p* < 0.05 was considered statistically significant). All data were included in statistical analysis with no randomization or blinding. No data points were excluded.

### 4.13. Ethics Statement

The research was approved by the ethics committee of the Medical Faculty of Kiel University (reference D 443/09). Written informed consent was obtained from all patients.

## 5. Conclusions

The present study provides evidence that the MCT1-mediated import of lactate into ”reverse Warburg“ PDAC cells not only supplies these cells with a substrate for energy production, but also with an efficient driver of metabostemness. This modality along with the reverse Warburg metabolism may essentially contribute to malignant traits such as therapy resistance. Targeting the MCT1 dependent lactate transport could be therefore a novel option in PDAC therapy, particularly by eliminating cancer stem cell-like cells deriving from metabolic restraints in the tumor and giving rise to recurrence upon conventional cancer therapy.

## Figures and Tables

**Table 1:** Preculture under glucose restriction plus lactate primes BxPc3 and T3M4 cells for accelerated cell cycle upon reseeding in normal medium. Both BxPc3 and T3M4 were precultured for 72h in normal medium containing 2 g/L glucose without (2 g) or with 20 mM lactate (2 g + L) or in low glucose medium either without (0.5 g) or with 20 mM lactate (0.5 g + L). Then, cells were reseeded in normal medium. The PI-cell cycle analysis was carried out (**A**) 48 h later or (**B**) before reseeding. The mean values ± SD from six inde­pendent experiments are shown (see also Figure 4C); *p*-values in panel (**A**) indicate statisti­cal significance between the data from pretreatment with low glucose plus lactate medium (0.5 + L) and data from pretreatment with normal medium (2 g) as highlighted in blue.

(**A**)	**BxPc3**	**G1 (%)**	**S (%)**	**G2/M (%)**
	**2 g**	57.37 ± 5.44	23.18 ± 5.21	19.46 ± 2.04
	**2 g + Lactate**	55.27 ± 4.18	23.75 ± 3.49	20.98 ± 4.83
	**0.5 g**	69.42 ± 8.22	16.47 ± 4.37	14.12 ± 4.75
	**0.5g + Lactate**	48.80 ± 5.44 (*p* < 0.01)	30.29 ± 5.03 (*p* < 0.02)	20.91 ± 4.56 (*p* > 0.1)
	**T3M4**	**G1 (%)**	**S (%)**	**G2/M (%)**
	**2 g**	53.74 ± 4.77	26.33 ± 3.72	19.93 ± 1.98
	**2 g + Lactate**	54.69 ± 4.57	26.80 ± 3.57	18.51 ± 1.32
	**0.5 g**	60.19 ± 6.56	20.94 ± 4.26	18.87 ± 2.49
	**0.5g + Lactate**	42.15 ± 4.03 (*p* < 0.001)	34.21 ± 1.95 (*p* < 0.003)	23.64 ± 2.31 (*p* < 0.002)
(**B**)	**BxPc3**	**G1 (%)**	**S (%)**	**G2/M (%)**
	**2 g**	55.25 ± 4.85	25.02 ± 7.12	19.73 ± 3.41
	**2 g + Lactate**	54.44 ± 6.77	23.38 ± 5.22	22.18 ± 3.60
	**0.5**	74.90 ± 5.32	11.82 ± 2.71	13.27 ± 4.38
	**0.5g + Lactate**	71.91 ± 8.07	13.39 ± 2.96	14.72 ± 4.75
	**T3M4**	**G1 (%)**	**S (%)**	**G2/M (%)**
	**2 g**	52,62 ± 3.24	27.69 ± 5.27	19.74 ± 2,40
	**2 g + Lactate**	54.54 ± 4.15	27.42 ± 4.78	18.05 ± 2.62
	**0.5 g**	78.62 ± 4.52	11.41 ± 2.66	9.97 ± 2.83
	**0.5 g + Lactate**	76.95 ± 3.53	11.98 ± 3.93	11.08 ± 2.77

## Supplementary Materials

The following are available online at https://www.mdpi.com/2072-6694/12/3/581/s1, Supplementary file: all original western blot figures.

## Abbreviations

CSC	cancer stem cell
KLF4	*Krüppel*-like factor 4
MCT1	monocarboxylate transporter 1
MCT4	monocarboxylate transporter 4
Oct4	Octamer binding transcription factor 4
PanIN	Pancreatic Intraepithelial Neoplasia
PDAC	pancreatic adenocarcinoma
PNI	perineural invasion
Sox2	sex determining region Y (SRY)- box 2
