# Supplementary material for: Impact of the Monocarboxylate Transporter-1 (MCT1)-Mediated Cellular Import of Lactate on Stemness Properties of Human Pancreatic Adenocarcinoma Cells
Source: Cancers (Basel). 2020 Mar 3;12(3):581. doi: 10.3390/cancers12030581 (PMC7139999; doi:10.3390/cancers12030581)
Supplement: Supplementary file 1 [file cancers-12-00581-s001.pdf]

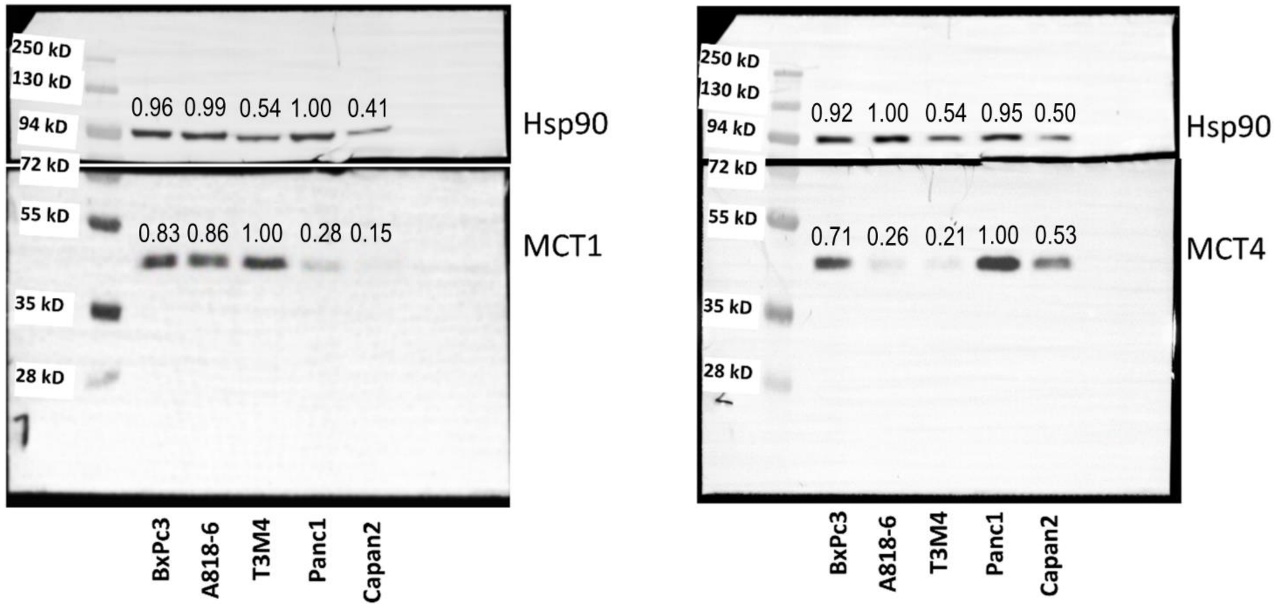

Figure 2A: original blots including size standard and band intensity ratios

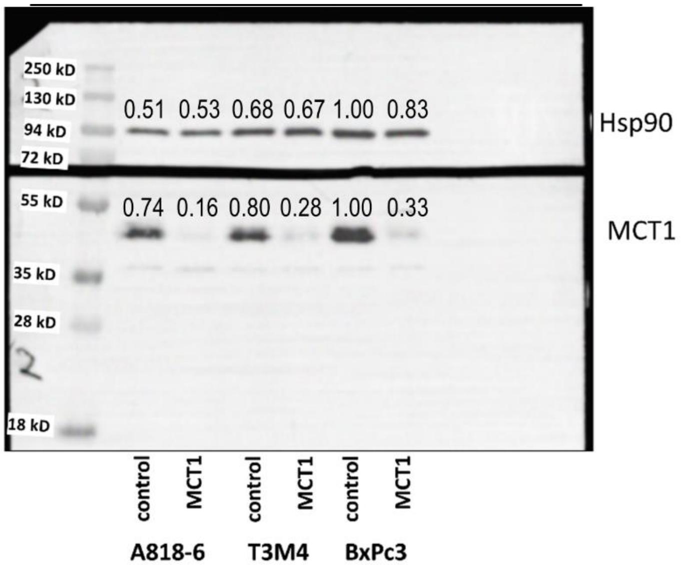

Figure 2C: original blots including size standard and band intensity ratios

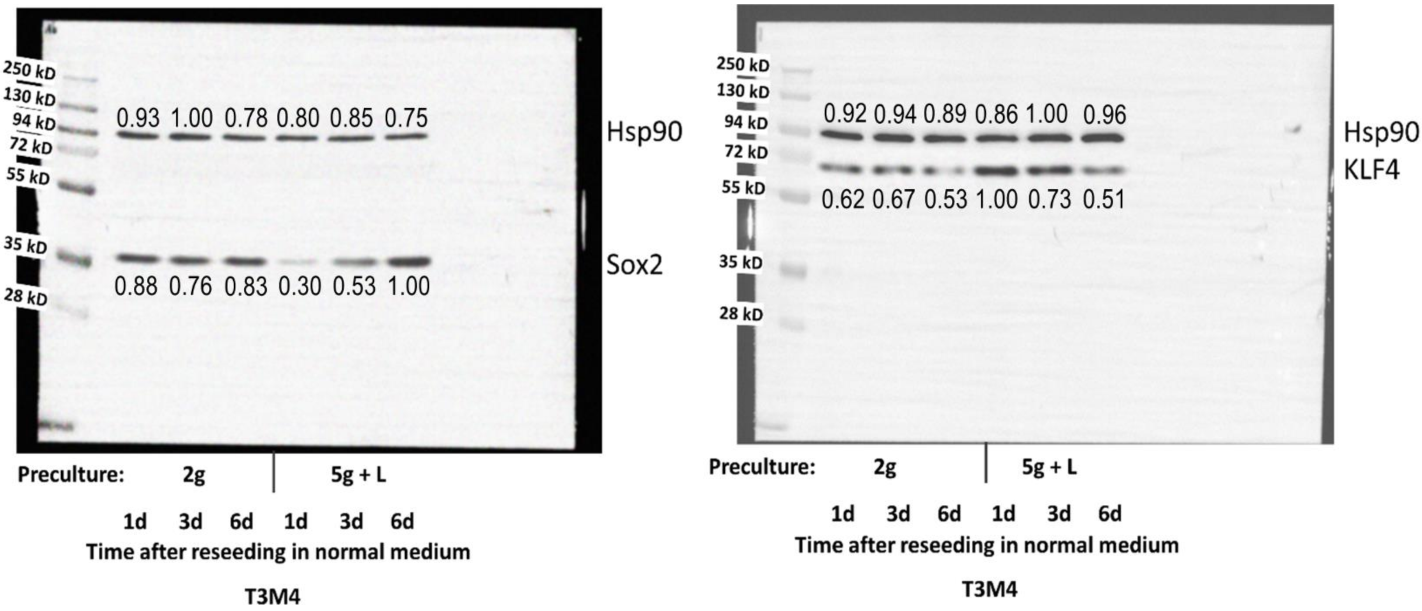

Figure 6D: original blots including size standard and band intensity ratios
